# Continuous video capture, and pollinia tracking, in *Platanthera* (Orchidaceae) reveal new insect visitors and potential pollinators

**DOI:** 10.7717/peerj.13191

**Published:** 2022-05-09

**Authors:** Genevieve E. van der Voort, Scott R. Gilmore, Jamieson C. Gorrell, Jasmine K. Janes

**Affiliations:** 1 Department of Ecosystem Science and Management, University of Northern British Columbia, Prince George, BC, Canada; 2 Biology, Vancouver Island University, Nanaimo, BC, Canada; 3 Unaffiliated, Lantzville, British Columbia, Canada; 4 Species Survival Commission, Orchid Specialist Group, North America, IUCN, Nanaimo, British Columbia, Canada

**Keywords:** Pollination, Raspberry Pi, Hybridization, Lepidoptera, Hymenoptera, Cameras, Biodiversity

## Abstract

Orchids often have specific pollinators, which should provide reproductive isolation, yet many produce natural hybrids. *Platanthera dilatata* and *P. stricta* differ in floral morphology but often co-occur, overlap in flowering, and are reputed parents of *P. ^x^estesii*. We used motion-triggered video detection units to monitor floral visitors of *P. dilatata* and *P. stricta* on Vancouver Island, Canada. Pollinia removal in *P. dilatata* was observed using histochemical staining, and cross-pollinations were performed to determine compatibility. From 1,152 h, 753 videos were recorded; 655 contained insects and 91 contained arachnids. Bumblebees, butterflies, and moths removed pollinia from *P. dilatata*. No pollinia removal was observed from *P. stricta*. Five videos showed insects moving between *Platanthera* species. Pollinia removal rates were low. Hand-pollinations resulted in capsule development and seed production. This study adds to the known diversity of insects interacting with these orchids, and highlights regional differences in floral visitors.

## Introduction

The relationship between plants and their pollinators is often vital for reproduction and can affect the ecology and evolution of species. Plant community composition may be limited by the availability of pollinators (Sargent & Ackerly, 2008), and different pollinators may drive the evolution of different floral characteristics (Boberg et al., 2014). If plant species have similar floral characteristics, they may attract similar pollinators and facilitate hybridization if other reproductive barriers are absent or weak (Argue, 2012a). For example, the pollination syndrome of many butterfly-attracting orchids in North America is often characterized by the following traits: nectar in a spur, open during the day, and red, orange, yellow, or blue colouration (Argue, 2012a). However, the generalization of pollination syndromes is debated (*e.g*., Fenster et al., 2004; Argue, 2012a).

Natural hybridization can have evolutionary and conservation impacts for both the parent species and the hybrid (Cozzolino et al., 2006; Jarvis et al., 2017; Cai et al., 2019). Introgression can lead to the loss of a parent species, potentially through outbreeding depression (Buerkle, Wolfe & Rieseberg, 2003) or genetic swamping (Todesco et al., 2016). However, hybridization may also lead to the creation of new species (Buerkle, Wolfe & Rieseberg, 2003; Abbot & Rieseberg, 2012), or enable populations to better adapt to changing environments (Chan, Hoffmann & van Oppen, 2018). Thus, hybridization has the potential to both aid in conservation efforts (*e.g*., evolutionary rescue) or hinder them (*e.g*., genetic erosion) (Vilà, Weber & Antonio, 2000; Parsons & Hermanutz, 2006; Genovart, 2008). For example, hybridization with an introduced *Celastrus*, facilitated by shared native bee pollinators, was considered a threat to the genetic integrity of the native *Celastrus scandens* (Zaya et al., 2021). Hence, filling knowledge gaps about species-specific factors in plant reproduction (*e.g*., pollinators and potential for hybridization) seems crucial to address these concerns.

The orchids (Orchidaceae) are one of the largest plant families with a wide biogeographic distribution (Christenhusz & Byng, 2016). Orchids are equally diverse in terms of their pollination strategies, which include self-pollination (Suetsugu, 2014), outcrossing using nectar rewards or deception (Argue, 2012a), trapping insects inside flowers (Pemberton, 2013), and sexual deceit of male insects (Paulus, 2019). Orchid pollination strategies often require the evolution of specialized floral morphology, such as nectar-containing spurs or a modified labellum resembling a female insect (Nilsson, 1992). As many orchid species exhibit some type of floral modification, the relationship orchids have with their pollinators is expected to be highly specialized to efficiently facilitate the movement of pollinia (Tremblay, 1992). However, the degree of orchid-pollinator specificity varies; several orchid species may rely on one or few pollinators (Tremblay, 1992), while other orchids are able to use a range of pollinators within a functional group (Patt et al., 1989; Fenster et al., 2004). While specialized pollinator relationships potentially provide reproductive isolation (Cozzolino & Scopece, 2008), natural hybridization is common in many orchid groups (Whitney et al., 2010), and shared pollinators, or pollinators within the same functional group, increase the likelihood of hybridization. For example, Schatz (2006) found that a natural hybrid (*Orchis* ^×^*bergonii*) was produced from *O. simia* and *O. anthropophora* when the shared pollinator (Coleoptera: *Cidnopus pilosus*) of the two parent species was present.

**Figure 1:**
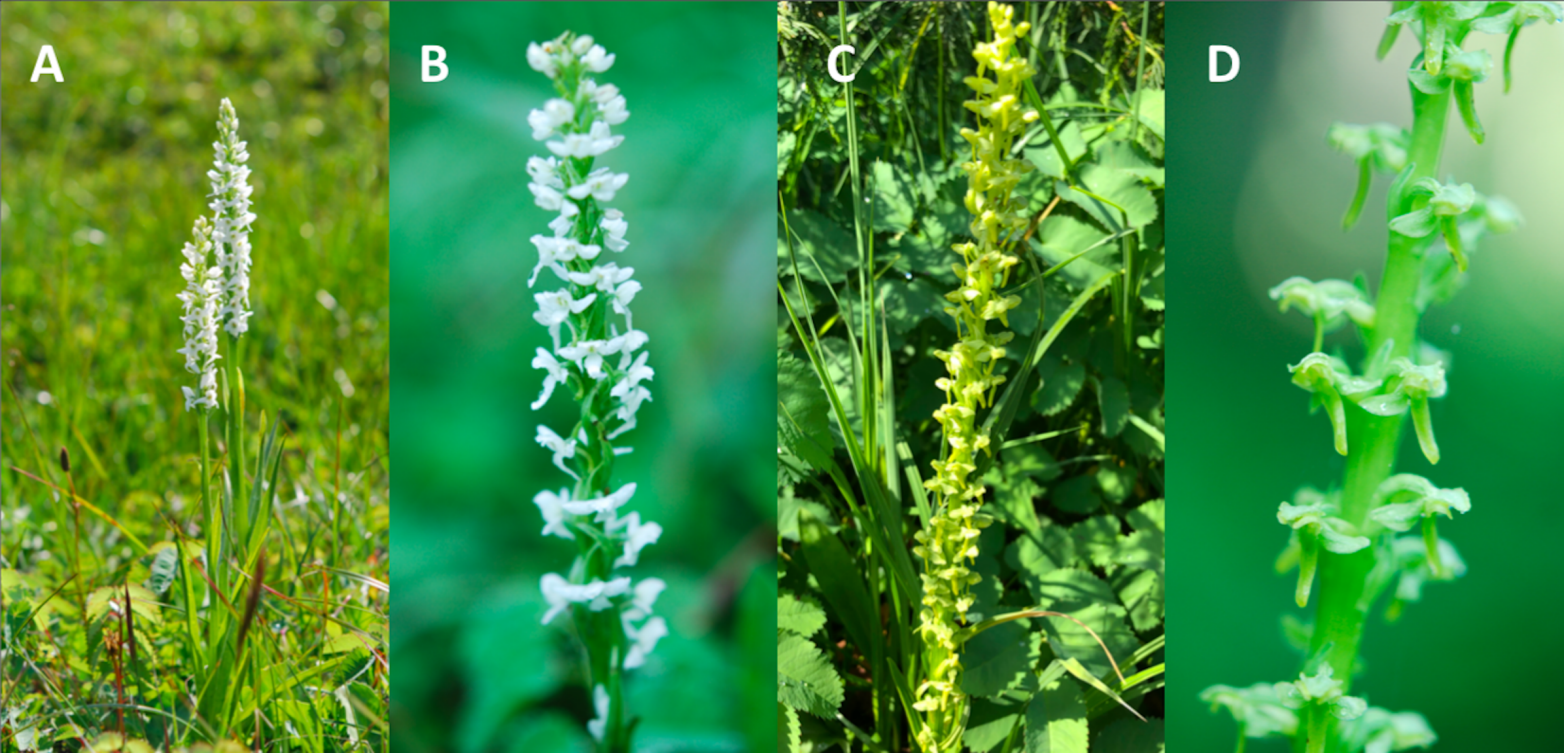
Representatives of the orchid species used in this study. (A and B) *Platanthera dilatata* (white bog orchid). (C and D) *Platanthera stricta* (slender bog orchid). Photo credit: Jasmine Janes.

The genus *Platanthera* Rich. (Orchidaceae) contains many species that are reported to hybridize (Brown, 2006; Argue, 2012b). The genus is widespread globally and many aspects of the pollination biology of European species have been examined, especially for *P. bifolia* and *P. chlorantha* (Nilsson, 1983; Maad & Nilsson, 2004; Steen, 2012; Boberg et al., 2014; Esposito et al., 2018). Two species within Section *Limnorchis*, *P. dilatata* (white bog orchid) and *P. stricta* (slender bog orchid), occur throughout North America, although *P. stricta* is restricted to western North America (Brown, 2006). Both species vary in terms of inflorescence colour, spur length, and scent (Argue, 2012b) (Fig. 1). *Platanthera dilatata* is strongly scented, has a white inflorescence, and spurs that range from 0.2–2 cm depending on the taxonomic variant (Brown, 2006; Argue, 2012b), whereas *P. stricta* has a green inflorescence, a shorter spur length (0.2–0.4 cm) (Brown, 2006; Argue, 2012b), and may (Patt, Rhoades & Corkill, 1988) or may not (Argue, 2012b) produce floral odour. Both species co-occur in estuarine, wetland, bog, and subalpine meadow areas (Brown, 2006; Argue, 2012b). Given the morphological differences between the two species, it has long been proposed that lepidopterans and short-tongued insects pollinate *P. dilatata* and *P. stricta*, respectively (Argue, 2012b). To date, *P. dilatata* pollinators include noctuid moths in Newfoundland, Canada (Boland, 1993), and Oregon, USA (Larson, 1992), and a hesperiid butterfly (Boland, 1993). In contrast, *P. stricta* pollinators include prodoxid and geometrid moths, empidid flies and mosquitoes, and halictid bees and bumblebees in Washington, USA (Patt et al., 1989). Despite the variation in reported pollinators, *P. dilatata* and *P. stricta* have been reported to create the hybrid *P*. ^×^*estesii* (Brown, 2006).

Aside from the few studies mentioned above, little is known about the parental species, and which pollinator(s) may be responsible for the hybridization. Several morphometric, molecular, and mycorrhizal investigations have been performed on *P. dilatata* in the USA (*e.g*., Wallace, 2003; Adhikari & Wallace, 2014). However, much of the ecology and reproductive biology is unknown, and both species remain understudied in Canada, particularly British Columbia. A previous study in British Columbia revealed low family-level diversity associated with habitats supporting *Platanthera* species (van der Voort, Saunders & Janes, 2021) but was limited to pan-trapping and timed observations. The objectives of this study were to: (1) identify and characterize floral visitors and pollinators of *P. dilatata* and *P. stricta* using continuous video monitoring, (2) identify any shared pollinators between *P. dilatata* and *P. stricta*, (3) determine rate and distance of pollinia transfer to fill some of the knowledge gaps surrounding these species, and (4) to confirm that cross-fertilization results in seed production. These data will contribute to a growing body of knowledge relating to biodiversity patterns in western Canada, potential differences in local floral visitors for widespread plant species, and generally increase our understanding of the plant-insect interactions these orchids support.

## Methods

### Continuous video monitoring units

Each continuous video monitoring unit consisted of a separate diurnal and nocturnal camera made from Raspberry Pi 3B+ boards (Raspberry Pi Foundation, Cambridge, United Kingdom) connected to Raspberry Pi V2 cameras (Raspberry Pi Foundation, Cambridge, United Kingdom) for diurnal use, and Raspberry Pi NoIR V2 cameras (Raspberry Pi Foundation, United Kingdom) for nocturnal use. Nocturnal cameras were fitted with BrightPi LED and IR lights (Raspberry Pi Foundation, Cambridge, United Kingdom). Units were powered by a 10,050 mAh (diurnal) or 12,000 mAh (nocturnal) power bank. BrightPi configuration scripts were edited to link the lights to the camera, and brightpi-control python scripts (https://github.com/raspberrycoulis/brightpi-control.git) operated the IR light pins independent of the LED ones. Lights were programmed to turn on automatically at a specific time using crontab commands.

We installed PiKrellCam motion detection software (https://billw2.github.io/pikrellcam/pikrellcam.html) with the following parameters: motion = on, confirm gap = 5, pre-capture = 5, event_gap = 10, post_capture = 2, video limit = 60, motion stills = off, max stills = 30, video resolution = 1,080. Thus, video was recorded, once motion was detected, for a minimum of one minute or until no motion had been detected for five seconds. Camera equipment was placed in clear waterproof plastic containers (Betty Crocker, 22 × 17 × 9 cm, side clip locks). For more detailed assembly instructions, refer to https://www.jasminejanes.com/post/making-raspberry-pi-in-the-noir-part-i. Video and thumbnail image files were stored on 256 Gb USB thumbdrives. Files were wirelessly transferred to external hard drives every time a battery change occurred.

Two continuous video monitoring units were deployed per site for 96 h at each site from late July to early August 2020 (*N* = 12). To minimize false motion triggers, surrounding vegetation was trimmed, black card was mounted behind the focal orchid plants, and floral wire was used to stabilize individual plants during wind gusts. Within a site, each unit was separated by a minimum of 3 m.

### Study sites for continuous video monitoring of orchids

We established three paired sites (*N* = 6) in the Strathcona Provincial Park area, Vancouver Island, British Columbia, Canada (Table 1), with each separated by a minimum of 50 m. The first pair of sites contained both *P. dilatata* and *P. stricta* in subalpine meadow habitat within Strathcona Provincial Park (Letter of Authorization 98700-20/Strathcona). The second represented *P. dilatata* in more disturbed areas – roadside ditches, and the third pair were located in *P. dilatata* patches in meadow habitats and roadsides subject to seasonal slashing. All *P. dilatata* specimens were likely *P. dilatata* var. *dilatata* based on the length of the floral spur being more or less equal to the length of the labellum. Temperature and humidity data were collected at each site every hour using Kestrel K2 Drops (Kestrel Instruments, Shawnee On Delaware, PA, USA).

**Table 1:** Summary information for study sites.

Site	Altitude (m)	Latitude	Longitude	Start date	End date	Estimated population size	Habitat type	Camera numbers	Number plants observed	
									***P. dilatata***	***P. stricta***
1	1,072	49.7361	−125.31441	21 July 2020	25 July 2020	50 *P. dilatata* 100 *P. stricta*	Alpine meadow	D1, N3	2	1
								D2, N2	2	
2	1,076	49.74361	−125.31886	21 July 2020	25 July 2020	100 *P. dilatata* (150 *P. stricta* within 10 m)	Alpine meadow	D4, N1	2	
								D3, N4	4	
3	1,027	49.7342	−125.27766	25 July 2020	29 July 2020	100 *P. dilatata* 20 *P. stricta*	Roadside	D4, N3	5	3
								D3, N1	2	
4	1,115	49.73144	−125.29999	25 July 2020	29 Jul 2020	30 *P. dilatata*	Roadside	D2, N2	2	
								D1, N4	4 (2 at night)	
5	1,074	49.75478	−125.32707	29 July 2020	02 August 2020	12 *P. dilatata*	Roadside	D3, N1	3 (1 during day)	
								D4, N3	1	
6	1,167	49.73814	−125.30368	29 July 2020	02 August 2020	20 *P. dilatata* (30 *P. stricta* within 20 m)	Slashed ditch	D1, N4	1	
								D2, N2	2	
Total plants observed									29	4

**Note:**

Due to the fixed position of separate diurnal and nocturnal cameras, some observed numbers may vary as indicated by numbers in parentheses.

### Assessments of pollinia removal and hybrid crosses

Five sites of *P. dilatata* were used to assess rates of pollen removal and transfer (Table 2). At each site, we applied approximately 0.5 ${\rm \mu }$l of six different histochemical stains (methylene aniline blue, orange G, neutral red, brilliant green, crystal violet, and rhodamine (Fisher Scientific, Ontario, Canada)) to individual plants using a 700 ${\rm \mu }$l Hamilton syringe (Fisher Scientific, Ontario, Canada). Each plant had 10 individual flowers’ worth of pollinia stained one colour. Each stain was applied at a concentration of 1% w/v, except for orange G which was applied at 10% w/v. This staining method has previously proven useful in tracking orchid pollinia in field situations (Peakall, 1989). As each flower contains two pollinaria, a total of 60 pollinia or 120 pollinaria were stained per site (total pollinaria: *N* = 600). Stained plants were monitored for three consecutive days to determine the number of pollinaria removed each day. We tracked stained pollinia, and the distance travelled, by inspecting all *P. dilatata* present within a site. Additionally, counts of non-stained pollinaria found on stained flowers were recorded.

**Table 2:** Rates of pollinia removal and transfer per site, per stain colour.

Site	Latitude	Longitude	Altitude (m)	Start date	Colour	Total pollinia removed	Total geitonogamous transfers	Total outcrossing transfers
Alpine Village*	49.73889	−125.30388	1,165	26 July 2021	Blue	3	0	0
					Red	3	0	0
					Orange	0	0	0
					Pink	9	0	0
					Purple	13	0	0
					Green	7	0	0
					Average	5.8 $\pm$ 1.9	0.0	0.0
Washington Way	49.73814	−125.30368	1,167	30 July 2021	Blue	6	0	1
					Red	5	1	1
					Orange	3	1	0
					Pink	7	2	0
					Purple	8	1	3
					Green	5	0	0
					Average	5.6 $\pm$ 0.7	0.8 $\pm$ 0.3	0.8 $\pm$ 0.5
Village Run	49.73964	−125.30428	1,153	30 July 2021	Blue	9	1	0
					Red	2	0	1
					Orange	4	1	1
					Pink	6	2	0
					Purple	9	0	0
					Green	4	0	0
					Average	5.7 $\pm$ 1.1	0.7 $\pm$ 0.3	0.3 $\pm$ 0.2
Alpine Resort 1*	49.74044	−125.30095	1,172	05 August 2021	Blue	2	0	0
					Red	4	2	0
					Orange	2	0	1
					Pink	0	0	0
					Purple	1	1	0
					Green	3	0	0
					Average	2.0 $\pm$ 0.6	0.5 $\pm$ 0.3	0.2 $\pm$ 0.2
Alpine Resort 2*	49.74006	−125.29881	1,172	05 August 2021	Blue	5	1	2
					Red	0	0	0
					Orange	0	0	0
					Pink	3	1	1
					Purple	0	0	0
					Green	4	1	0
					Average	2.0 $\pm$ 0.9	0.5 $\pm$ 0.2	0.5 $\pm$ 0.3

**Note:**

Asterisks indicate that there was rain during the observation period.

At one site, five flowers on each of three *P. dilatata* plants were hand-pollinated with *P. stricta* pollinia from three separate plants. Crosses were only performed in this direction as it was toward the end of the *P. stricta* flowering period and healthy, unpollinated flowers were scarce. Hand-pollinated flowers were tagged, seed capsule development monitored, and capsules collected in October 2020.

### Data analysis

Insects in videos were identified to taxonomic family or lower, and behavioural observations recorded: flying by the orchid, on a flower (including if the insect landed on or flew off the flower), on a plant (including if the insect landed on or flew off the plant), hovering near a flower, active on a flower (*e.g*., using its proboscis to feed from the flower), and other (*e.g*., sitting on the background paper). For each insect present in a video file, the following were recorded: the number of visits to individual plants, the number of individual flowers visited per plant, duration of each visit, starting times, end times, and total length of the video. Because many insects would quickly fly by the plant, we performed lower-level identifications on the videos that showed insects directly interacting with the plant, and particular attention to those insects seen removing pollinia. Sunrise and sunset times were approximately 05:47 and 20:55, respectively, during our study period. We placed video files into one of four, 6-h time bins, to better identify preferred insect activity periods: (1) dawn = 00:01 to 06:00; (2) morning = 06:01 to 12:00; (3) afternoon = 12:01 to 18:00, and (4) dusk = 18:01 to 24:00.

We calculated the mean rate of pollinia removal within and among sites, and the mean transfer distance. Rates of removal per stain colour were also assessed. Differences in mean insect activity levels across four time periods, pollinia removal among sites, and pollinia removal among stain colours were compared using a Kruskal-Wallis nonparametric analysis of variance with a Dunn *post hoc* test, or a Conover *post hoc* test (pollinia removal only), after applying a Bonferroni correction. All analyses were conducted in R version 4.1.1 (R Core Team, 2021) with packages: FSA: Simple Fisheries Stock Assessment Methods (Ogle et al., 2021), multcompView (Graves et al., 2019), lsmeans (Lenth, 2016), and conover.test: Conover-Iman test of multiple comparisons using rank sums (Dino, 2017).

## Results

### Raspberry Pi continuous video monitoring units

The Raspberry Pi units performed well in a range of temperatures (1.1–41.6 °C) from 21 July 2020 to 02 August 2020, and were not damaged by rainfall events from 22–24 July 2020. The 10,050 mAh batteries lasted approximately 6–8 h, depending on the number of motion-trigger events, while the 12,000 mAh batteries exceeded 10 h of use. We found the motion-trigger to be sensitive enough to detect very small insects, such as mosquitos and ants, and the quality of the video files sufficient for identification to family-level or lower when insects landed on the plants.

### Continuous video monitoring of orchids and supplementary insect collection

*Platanthera stricta* was directly present, or nearby, at four study sites and *P. dilatata* was present at all six sites (see Table 1). The mean daily (6:00–21:00) temperature during the study was 18.4 °C (SE = ±0.4) with 69.8% (SE = ±1.3) relative humidity, while nocturnal (21:00–5:00) mean temperature was 9.5 °C (SE = ±0.2) with 95.1% (SE = ±0.5) relative humidity. We recorded 753 motion-triggered videos over a combined 1,152 h of observation from 12 cameras and six study sites. Mean video length was 16 (SE = ±9) seconds, and the combined length of all videos was 3:27:39 h, while the mean start time was 14:11:29 h (SE = ±4:21:27). The afternoon time category had the highest number of triggered videos (*N* = 335), followed by morning (*N* = 227), dusk (*N* = 176), and dawn (*N* = 15). We found significant differences in the mean number of videos recorded across the four time categories (H(3) = 77.57, *p* < 0.001) with afternoon (mean = 3.3 ± 0.5) having significantly more videos than dawn (mean = 0.1 ± 0.1, *p* < 0.001) and dusk (1.7 ± 0.3, *p* < 0.05), but not the morning (2.2 ± 0.4, *p* > 0.10). The dawn period had fewer videos than morning (*p* < 0.05) and dusk (*p* < 0.05), though there was no difference between morning and dusk (*p* > 0.10). Only one nocturnal video showed an insect interacting with an orchid (active, landing/leaving, or hovering) between 21:01–05:41, compared to 260 diurnal interaction videos (05:42–21:00).

**Figure 2:**
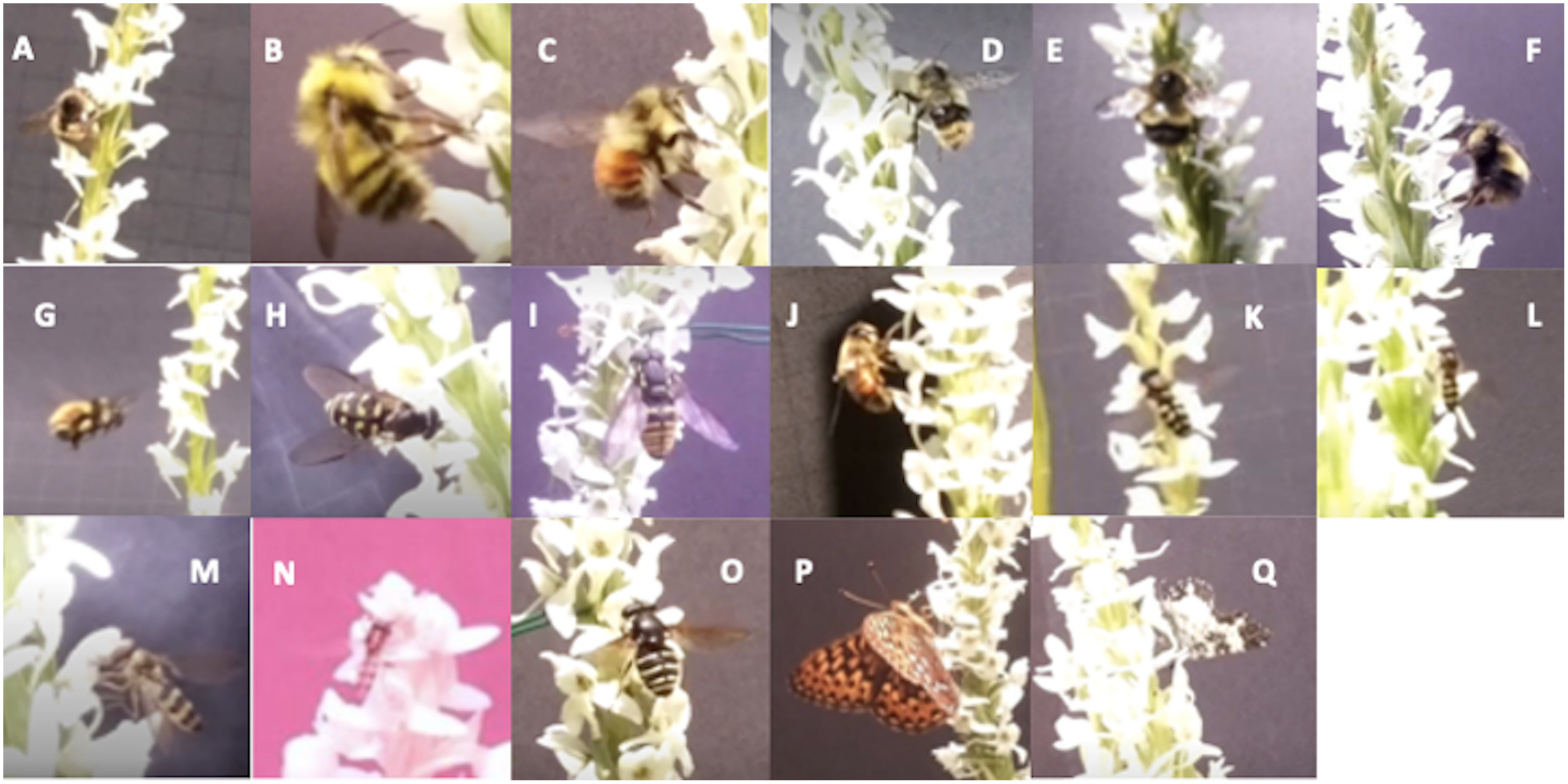
Panel of identified insects that directly interacted with orchid species. (A and B) *Bombus flavifrons*; (C) *Bombus melanopygus*; (D) *Bombus mixtus*; (E and F) *Bombus sitkensis*; (G) *Bombus vancouverensis*; (H) *Dasysyrphus intrudens*; (I) *Chrysotoxum* sp.; (J) *Eristalis* sp.; (K) *Eupeodes* sp.; (L) *Lapposyrphus lapponicus*; (M) *Meliscaeva cinctella*; (N) *Platycheirus* sp.; (O) *Sericomyia chalcopyga*; (P) *Speyeria hydaspe*; (Q) *Rhuemaptera* sp. Image credit: Genevieve van der Voort and Jasmine Janes.

We observed insects in 655 video files, and arachnids in 91, across the 753 total videos. Insects comprised four taxonomic orders, 11 families, two subfamilies, 12 genera, and 10 species (Table 3). Flying by the plant was the most common insect behaviour recorded in 282 videos, while landing on a flower was the most frequently recorded behaviour involving direct interaction with the orchid for both *Platanthera* species (Table 3). Some insect observations went over several video files due to pauses in activity. We obtained 207 unique observations of insects interacting with *P. dilatata* and 17 for *P. stricta*. However, the motion trigger rate per plant (number of videos/number of plants) was 7.1% for *P. dilatata* and 4.3% for *P. stricta*. Species-level identification was possible for 33% of these videos. Sixteen insects were seen removing orchid pollinia from *P. dilatata*; pollinia became attached to their antennae or proboscis. The majority of these removals were from *Bombus* spp. bumblebees (*N* = 14: nine *B. flavifrons*, one *B. mixtus*, one *B. sitkensis*, and three unidentified *Bombus* spp.) (Fig. 2). One fritillary butterfly (Nymphalidae: *Speyeria hydaspe*; Fig. 2; see Sup. Mat. 1 for video) and one noctuid moth were observed with *P. dilatata* pollinia attached to their proboscises. We did not observe any pollinia removal from *P. stricta*. Five videos showed insects moving between both *P. dilatata* and *P. stricta*: two with syrphid flies that hovered near, or landed on, flowers of both species, and three showed pterophorid moths landing on both species. Of these, only one pterophorid appeared to probe inside flowers from both species, starting on *P. dilatata* then moving to *P. stricta*; however, no pollinia removal or transfer could be seen.

**Table 3:** Identification information for insects interacting with *P. dilatata* and *P. stricta*.

*Platanthera* spp. interacted with	Behaviour	Order	Family	Subfamily	Genus/species
*P. dilatata* (207)	Active on flower(s) (45)	Diptera (12)	Syrphidae (12)	Syrphinae (8)	*Lapposyrphus lapponicus* (1)
					*Eupeodes* sp. (1)
				Eristalinae (2)	*Eristalis* spp. (2)
		Hymenoptera (30)	Apidae (30)	Apinae (30)	Bombus flavifrons* (10)
					*Bombus melanopygus* (7)
					Bombus mixtus* (1)
					Bombus sitkensis* (3)
					*Bombus* spp.* (9)
		Lepidoptera (3)	Geometridae (1)	Larentiinae (1)	*Rheumaptera* sp. (1)
			Noctuidae * (1)		
			Nymphalidae (1)	Heliconiinae (1)	Speyeria hydaspe* (1)
	Landed on/flew off flower (86)	Coleoptera (1)	Staphylinidae (1)		
		Diptera (49)	Syrphidae (41)	Syrphinae (39)	*Chrysotoxum* sp. (1)
					*Dasysyrphus intrudens* (complex) (1)
					*Meliscaeva cinctella* (3)
					*Platycheirus* (2)
				Eristalinae (1)	*Sericomyia chalcopyga* (1)
			Unidentified Diptera (8)		
		Hymenoptera (24)	Apidae (22)	Apinae (22)	*Bombus flavifrons* (3)
					*Bombus melanopygus* (9)
					*Bombus* spp. (10)
			Formicidae (2)	Formicinae (1)	*Campanotus* sp. (1)
		Lepidoptera (6)	Pterophoridae (6)		
		Unidentified Insect (6)			
	Hovered near flower(s) (66)	Diptera (48)	Bombyliidae (1)		
			Syrphidae (45)	Syrphinae (44)	*Lapposyrphus lapponicus* (1)
					*Platycheirus* sp. (1)
			Unidentified Diptera (2)		
		Hymenoptera (18)	Apidae (17)		*Bombus melanopygus* (3)
					*Bombus* *vancouverensis* (1)
					*Bombus* spp. (13)
			Vespidae (1)		
	Landed on/flew off other areas of plant (10)	Diptera (6)	Syrphidae (3)		
			Tipulidae (1)		
			Unidentified Diptera (2)		
		Hymenoptera (2)	Formicidae (2)		
		Unidentified Insect (2)			
*P. stricta* (17)	Active on flower(s) (1)	Lepidoptera (1)	Pterophoridae (1)		
	Hovered near flower(s) (1)	Diptera (1)	Syrphidae (1)	Syrphinae (1)	
	Landed on/flew off flower (12)	Diptera (3)	Syrphidae (3)	Syrphinae (3)	
		Lepidoptera (5)	Pterophoridae (5)		
		Unidentified Insect (4)			
	Landed on/flew off other areas of plant (3)	Lepidoptera (2)	Pterophoridae (2)		
		Unidentified Insect (1)			
*P. dilatata* and *P. stricta* (4)	Active on flower(s) (1)	Lepidoptera (1)	Pterophoridae (1)		
	Landed on/flew off flower (2)	Lepidoptera (2)	Pterophoridae (2)		
	Hovered near flower(s) (1)	Diptera	Syrphidae (1)	Syrphinae (1)	
No orchid interaction (279)	Flew by	Diptera (114)	Syrphidae (84)		
			Tipulidae (1)		
			Unidentified Diptera (29)		
		Hymenoptera (59)	Apidae (58)	Apinae (57)	*Bombus* spp. (57)
			Vespidae (1)		
		Lepidoptera (1)	Pterophoridae (1)		
		Unidentified Insect (105)			

**Note:**

Numbers in parentheses indicate total insect observations for each taxonomic group. Asterisks indicate which insect groups were observed with orchid pollinia attached to them.

### Assessments of pollinia removal and hybrid crosses

We stained 10 flowers on six plants per site (10 flowers × 6 plants × 5 sites = 300 plants) resulting in 600 stained pollinaria. We observed 25 plants (83%) with stained pollinia removed, representing a total of 80 flowers (27%). In contrast, 12 of the stained plants (40%) received stained pollen, of any colour, across a total of 18 flowers (6%), and a total of eight unstained neighbours received stained pollinia. Thus, removal rates were considerably higher than deposition rates. At the site level, all plants at Washington Way and Village Run showed removals, whereas Alpine Resort 2 had the fewest plants (50%) exhibiting removal. When considering total numbers of flowers across plants, Alpine Valley and Village Run had the highest removal rates (22 each; 37%), while Alpine Resort 2 had the lowest (8 flowers; 13%).

When counting stained pollinia, removal was highest at Alpine Valley (29%, mean = 4.7, SE = ±1.9) and lowest at Alpine Resort 2 site (10%, mean = 2.0, SE = ±0.9), while the mean number of pollinia removed across all sites was 22% (mean = 4.2, SE = ±0.5). The mean pollinia removed varied significantly across all five sites (H(4) = 10.51, *p* = 0.03); however, both the Dunn and the Conover *post hoc* tests failed to differentiate among pairwise comparisons after accounting for Bonferroni corrections. Omitting a Bonferroni correction suggests that mean pollinia removal was higher in the three sites stained at the end of July compared to the two sites that were stained one week later but this pattern also coincides with rainfall events. Alpine Village received rain on day three of observations (29 July 2020), while Alpine Resort 1 and 2 received rain on day two (07 August 2020).

Purple stain had the highest average pollinia removal rate across all sites (31%, mean = 6.2, SE = ±2.5), while orange had the least removal (9%, mean = 1.8, SE = ±0.8). However, the mean number of pollinia removed did not significantly vary among stain colours (H(5) = 5.9, *p* > 0.3). Rates of pollinia removal suggestive of geitonogamy (*e.g*., red pollinia moved within the red-stained plant) were 3% (mean = 0.5; SE = ±0.1), and 2% (mean = 0.3; SE = ±0.1) for outcrossing (*e.g*., stained pollinia located on an unstained plant). In total, there were 11 incidences of stained pollinia found on unstained plants, with a mean transfer distance of 0.74 m (SE = 0.2). Table 2 provides a summary of the pollinia removal patterns observed by site and stain colour. All hand-pollinations, in which pollinia were transferred from *P. stricta* to *P. dilatata*, resulted in capsule development and seed production.

## Discussion

This study is the first to examine pollinators, and pollen movement, of *Platanthera dilatata* and *P. stricta* on Vancouver Island, British Columbia, Canada. It is also the first, to our knowledge, to use continuous video monitoring to observe plant-insect interactions for these orchid species. During our observations, *P. dilatata* was in early to peak flowering, while many of the *P. stricta* flowers were already at anthesis. Thus, we performed observations on 29 *P. dilatata* and four *P. stricta*. Four orders, 11 families, two subfamilies, 12 genera, and 10 species were observed from 753 insect videos. We observed three insect families removing pollinia from *Platanthera dilatata*: 14 individual *Bombus* spp. bumblebees (nine *B. flavifrons*, one *B. mixtus*, one *B. sitkensis*, and three unidentified *Bombus* spp.), one nymphalid butterfly (*Speyeria hydaspe*), and one unidentified noctuid moth. One video began with one *Bombus* spp. having pollinia attached to it, and was not included in the above total. Overall, we observed relatively high rates of pollinia removal from the stained plants (83%) but, considering the multi-flowered structure of the inflorescence, removal rates across flowers were relatively low (27%). Further, the rate of pollinia deposition was much lower (12 stained and eight unstained plants received stained pollinia).

### Continuous video monitoring units

Several studies have used timed flash photography (*e.g*., Suetsugu & Hayamizu, 2014; Steen, 2017) or video cameras such as GoPro (*e.g*., Steen & Mundal, 2013; Gilpin, Denham & Ayre, 2017; Lahondère et al., 2020) to successfully observe plant-insect interactions. However, we found these methods unsuitable for our study. Timed photography provides limited information, observations of behaviour are less likely, and the flash may inadvertently impact further insect activity. GoPro cameras, and other video recorders, are relatively expensive and require more modification to perform nocturnal observations, and in some cases, weatherproofing. Steen (2012) devised an effective nocturnal system for monitoring orchids but the specific type of camera used could not be sourced by us. We found the continuous video monitoring units used here to be cost effective (~CAD $250 ea), weatherproof, functional in both day and night settings, sensitive enough for small insect motion-triggers, and to have high enough resolution that family-level, or lower, taxonomic identification is often possible. Thus, the quality of our data seems comparable to that of others using similar setups (*e.g*., Droissart et al., 2021).

### Continuous video monitoring of orchids

Lepidopterans have been reported as the main pollinators of *Platanthera dilatata* (Argue, 2012b). For example, Larson (1992) observed individuals of *Anarta oregonica* (syn. *Discestra oregonica*) noctuid moths visiting *P. dilatata* in Oregon, USA, at around 15:00, with many having pollinia attached to their proboscises. In Newfoundland, Canada, Boland (1993) observed several noctuid moths within a 2-h window after dusk. In contrast, we observed a single noctuid moth at 04:48. All other active insects were recorded from 08:44 to 20:11. These differences may be explained by variation in noctuid species, abundance or behaviour between eastern and western Canada, or temperatures that were too cold for moths to be active. Insect biodiversity surveys appear sparse for Vancouver Island, particularly the Mt. Washington area, but several range maps suggest there may be local and regional differences in species richness and abundance on Vancouver Island compared to other parts of western and eastern North America. With respect to temperature, Boland (1993) observed moths flying at 12 °C, whereas our mean night temperature was 9.5 °C (SE = ±0.2). It is possible that the infra-red lights affected nocturnal insect behaviour (Callahan, 1977; Owens & Lewis, 2018; Macgregor & Scott-Brown, 2020). Flower density may also have an impact on insect visitation (Duffy & Stout, 2008; Essenberg, 2012). Larson (1992) reported that over 500 individuals of *P. dilatata* were present in clusters of around 25 individuals when 15–20 noctuids were observed. In contrast, populations of *P. dilatata* in our study had between 12–100 individuals. However, it is unknown how pollinator visitation relates to floral density within *Platanthera*, or if there are regional differences in nectar or scent composition that may impact visitation rates. Further studies identifying insect species in the area, and the impacts of floral density and chemistry, would be useful in determining why we observed so few noctuids.

Boland (1993) recorded a fritillary butterfly (*Boloria selene terraenovae*) visiting *P. dilatata* in Newfoundland, but it was not confirmed as a pollinator. The fritillary recorded here, identified as *Speyeria hydaspe*, was observed probing several flowers and removing pollina, which then attached to its eyes or the proximal end of its proboscis. Boland (1993) also observed a papilionid floral visitor and a hesperiid butterfly pollinator on *P. dilatata*. *Papilio* have been observed as floral visitors of *P. dilatata* in the Canadian Rockies (Catling & Catling, 1991; Argue, 2012b) but it is unclear if *Papilio* were confirmed as pollinators. Additionally, a geometrid moth appeared to be active within flowers of *P. dilatata* from 14:13 to 14:19 in our study. However, no pollinia removal was observed. Again, the different species and behaviours we observed highlight how little we know about *Platanthera* pollination ecology and their associated plant-insect community networks. These differences also remind us that widespread plant species can exhibit regional variation in insect visitation, as noted by Van der Niet, Peakall & Johnson (2014). Thus, observations across a species’ range are valuable.

The remaining insects observed with pollinia in our study were *Bombus* spp. (*B. flavifrons*, *B. mixtus*, *B. sitkensis*, and other unidentified *Bombus* spp.) between 09:57 and 18:26 on *P. dilatata*. Boland (1993) observed *Bombus* spp. visiting *P. dilatata* in Newfoundland, Canada; each bumblebee was active for under 20 s and none were confirmed as pollinators. Only three of the 14 bumblebees observed with pollinia were active on *P. dilatata* flowers for >20 s in our study (range = 1–81 seconds; mean = 19.7, SE = ±6.0 s). Watching our videos frame-by-frame shows pollinia being transferred to the glossa and labrum area in all instances. Interestingly, Patt et al. (1989) observed *Bombus* sp. as pollinators of *P. stricta* in Washington, USA, with insects probing a flower for 2–5 s, yet we observed no *Bombus* sp. interacting with *P. stricta*. Further, Patt et al. (1989) identified several other short-tongued insects as pollinators of *P. stricta* including prodoxid and geometrid moths, and syrphid and empidid flies. We only observed geometrids, syrphids, and bumblebees interacting with *P. dilatata*. Only plume moths (Pterophoridae) appeared to be active within the flowers of *P. stricta* in this study, and no plume moth was observed with, or removing, pollinia from *P. stricta*. Another *Platanthera* species, *P. unalascensis*, has been reported to have plume moths as pollinators in Sierra Nevada, USA (Kipping, 1971). Both *P. stricta* and *P. unalascensis* have similar floral characteristics: a green inflorescence and relatively short spurs (Brown, 2006). Continued observations of *P. stricta* starting earlier in their flowering season, and at sites with higher densities, would be useful to investigate the role pterophorid moths could have in *P. stricta* pollination.

Only five insects were observed moving between both *P. dilatata* and *P. stricta* in this study, and out of these five, only one pterophorid moth was observed active within flowers. No pollinia were visible on the moth. The hybrid of *P. dilatata* and *P. stricta*, *P*. ×*estesii*, is poorly documented and there is very little known about its range and origins. Brown (2006) reports that the hybrid is present in Olympic National Park in Washington, USA. We believe we documented a *P*. ×*estesii* individual on Mt. Washington, Vancouver Island, Canada, but the identity has not been confirmed from the available photographs (posted on iNaturalist). Hybridization between *P. dilatata* and *P. stricta* is likely achieved through a shared pollinator but details of the species and mechanisms involved are unknown. Due to differences in morphological and scent characteristics, representing different pollination syndromes, these two orchids should be pollinated by different insect species or functional groups (van der Cingel, 1995; Argue, 2012b), which would be consistent with the insects that have, thus far, been cited as pollinators (Patt et al., 1989; Boland, 1993; Larson, 1992). However, *P. dilatata* and *P. stricta* do co-occur, and overlap in pollinators, while presumably infrequent, may be possible.

Brown (2006) states that *P. dilatata* and *P. stricta* flower between late June and August, providing considerable overlap in flowering that may facilitate hybridization through shared pollinators. However, we observed limited overlap in flowering at Mt. Washington in the summer of 2020. Many of the *P. stricta* individuals were in the late stages of flowering, with lower flowers already at anthesis, whereas the *P. dilatata* were in the early to peak flowering period. This offset in flowering resulted in low numbers of *P. stricta* being filmed, low numbers of insects observed interacting with *P. stricta*, and low numbers of insects moving between *P. dilatata* and *P. stricta*. Thus, we interpret our results for *P. stricta*, and shared insect visitors with *P. dilatata*, with caution. Interestingly, in 2019 when we performed pan-trapping surveys in the area (van der Voort, Saunders & Janes, 2021) we observed considerable overlap in the flowering seasons between *P. dilatata* and *P. stricta*. Future studies should aim to capture insect interactions with *P. stricta* earlier in the flowering season. Additionally, these observations should be conducted over subsequent years, with the collection of environmental data (*e.g*., precipitation, winter temperature, etc.), in order to better assess which insect species visit *P. stricta*, and how often the two species’ flowering phenology overlaps.

### Assessments of pollinia removal and hybrid crosses

Peakall (1989) reported a removal rate of just 7% in *Prasophyllum fimbria* with a mean of 22% of pollinia transfers representing a geitonogamous (same plant, different flower) transfer. In contrast, we observed a geitonogamous transfer rate of 3% and an outcrossing rate of just 2%. However, these numbers are not directly comparable due to differences in data collection. For example, we recorded the movement of stained pollen to unstained neighbours for outcrossing, whereas Peakall (1989) recorded the numbers of unstained pollen being transferred to the stained plants. Additionally, Peakall (1989) stained all pollinia on the 16 experimental plants, whereas we stained 10 flowers worth of pollinia per plant. Levels of geitonogamous transfer may have been higher in our study because we could not differentiate among unstained pollinia from neighbours and some of the focal plant flowers. Thus, our geitonogamous transfer rate is conservative because we could only track the stained proportion of pollinia. Peakall (1989) also observed that the average transfer distance of 8 m was less than the average flight distance of bees and wasps suspected as pollinators. In contrast, our average transfer distance was <1 m. Our removal rates across flowers (27%) are also lower than those reported by Chen et al. (2021) for a *Habenaria*. Chen et al. (2021) found 75% of flowers had at least one pollinium removed in their study, and 83% of flowers received pollinia. However, they were not staining pollinia so there is no information on geitonogamous transfer rates.

*Habenaria, Platanthera* and *Prasophyllum* all represent nectar-rewarding species. Both *Habenaria* and *Platanthera* offer nectar in a spur, whereas *Praspophyllum* offers nectar droplets from secretory structures on the adaxial surface of the labellum. Thus, the types of pollinators attracted differs. For example, *Prasophyllum* are believed to pollinated by Hymenoptera (Peakall, 1989; Coates, Lunt & Tremblay, 2006), while many species of *Habenaria* and *Platanthera* are believed to be pollinated by Lepidoptera (Argue, 2012b). It is likely that the foraging behaviours of the pollinators contribute to the differences observed in pollinia removal rates and transfer distances. However, the pollinia removal differences among *Habenaria* and *Platanthera*, which are superficially similar in floral morphology, coupled with the range of floral visitors we observed on *Platanthera dilatata*, suggest the following: (1) that *P. dilatata* does not have strong pollinator fidelity; (2) that floral visitors are frequently removing pollinia but rarely transferring it for actual pollination; and/or (3) that the pollinator(s) are relatively ineffective. We did observe a number of insects engaging in pollinia removal but few transfers to the column of the flower were seen. More observations of the insect behaviours and morphology, and more sophisticated pollinia tracking methods would be required to determine why *P. dilatata* pollinia removal rates are higher than transfer/deposition rates, and what this means for population dynamics long-term.

As previously mentioned, *P. dilatata* and *P. stricta* differ in morphology, and likely scent. *Platanthera dilatata* has a white inflorescence and long spurs (0.2–2 cm depending on the taxonomic variant (Brown, 2006; Argue, 2012b)), while *P. stricta* has a green inflorescence and short spurs (0.2–0.4 cm) (Brown, 2006). Further, *P. dilatata* is described as being strongly scented, while *P. stricta* is believed to mostly produce aromatic compounds without a scent (Argue, 2012b). Many orchid species rely on visual and chemical attractants for pollinators, with chemicals attracting insects at a longer distance and visual attractants once the insect is closer (Vereecken, Dafni & Cozzolino, 2010; Van der Niet, Peakall & Johnson, 2014; Phillips et al., 2015). As it is unknown if pollinators of these two orchids are drawn to visual or chemical attractants, dissimilarities in colour, scent production, and spur length may have little effect on any shared pollinator(s). Further, which taxonomic variant of *P. dilatata* can act as the parent species in hybridization is unclear. Pollinia from three *P. stricta* individuals were transferred by hand to three *P. dilatata* var. *dilatata* individuals, resulting in capsule and seed development in all instances. However, the viability of the seeds is currently unknown.

## Conclusion

We used motion-triggered continuous video monitoring units to identify floral visitors and potential pollinators of *P. dilatata* and *P. stricta* in both nocturnal and diurnal settings. While we did not identify a shared pollinator among *P. dilatata* and *P. stricta*, we did observe a shared floral visitor, and it is possible that the phenology of flowering between the two species is more compatible in certain years. Thus, future studies are encouraged to better examine potential shared pollinators throughout the orchids’ flowering periods, as well as observing insect visitors to *P. ×estesii*. We also contribute to the known floral visitors of *P. dilatata*. Our observations suggest that *P. dilatata* on Mt. Washington, Vancouver Island, Canada, may be pollinated by a noctuid moth, a nymphalid butterfly (*Speyeria hydaspe*), and *Bombus* spp. (specifically *B. flavifrons*, *B. mixtus*, and *B. sitkensis*). Our study helps in highlighting the overall diversity of insects interacting with *P. dilatata*, and how the known visitors of a widespread plant can vary over regional scales. Considering that relatively few investigations of *Platanthera* pollination ecology have been conducted in North America, particularly Canada, this study contributes valuable knowledge that will be useful for future experiments and conservation work.

## Supplemental Information

10.7717/peerj.13191/supp-1 Supplemental Information 1 Example of the video obtained from the Raspberry Pi units. Video shows *Speyeria hydaspe* probing *Platanthera dilatata* flowers, and removing pollinia.

10.7717/peerj.13191/supp-2 Supplemental Information 2 Raw data for insect observations obtained from continuous video monitoring units, and pollinia staining experiments.

## Additional Information and Declarations

### Field Study Permissions

The following information was supplied relating to field study approvals (*i.e*., approving body and any reference numbers): Strathcona Provincial Park (Letter of Authorization 98700-20/Strathcona).

### Data Availability

The following information was supplied regarding data availability: The raw data is available in the Supplemental Files.
